# How Early Can the Seeding Dates of Spring Wheat Be under Current and Future Climate in Saskatchewan, Canada?

**DOI:** 10.1371/journal.pone.0045153

**Published:** 2012-10-10

**Authors:** Yong He, Hong Wang, Budong Qian, Brian McConkey, Ron DePauw

**Affiliations:** 1 Semiarid Prairie Agricultural Research Centre, Agriculture and Agri-Food Canada, Swift Current, Saskatchewan, Canada; 2 Department of Soil and Water Sciences, China Agricultural University, Beijing, China; 3 Eastern Cereal and Oilseed Research Centre, Agriculture and Agri-Food Canada, Ottawa, Ontario, Canada; Pacific Climate Impacts Consortium, Canada

## Abstract

**Background:**

Shorter growing season and water stress near wheat maturity are the main factors that presumably limit the yield potential of spring wheat due to late seeding in Saskatchewan, Canada. Advancing seeding dates can be a strategy to help producers mitigate the impact of climate change on spring wheat. It is unknown, however, how early farmers can seed while minimizing the risk of spring frost damage and the soil and machinery constraints.

**Methodology/principal findings:**

This paper explores early seeding dates of spring wheat on the Canadian Prairies under current and projected future climate. To achieve this, (i) weather records from 1961 to 1990 were gathered at three sites with different soil and climate conditions in Saskatchewan, Canada; (ii) four climate databases that included a baseline (treated as historic weather climate during the period of 1961–1990) and three climate change scenarios (2040–2069) developed by the Canadian global climate model (GCM) with the forcing of three greenhouse gas (GHG) emission scenarios (A2, A1B and B1); (iii) seeding dates of spring wheat (*Triticum aestivum* L.) under baseline and projected future climate were predicted. Compared with the historical record of seeding dates, the predicted seeding dates were advanced under baseline climate for all sites using our seeding date model. Driven by the predicted temperature increase of the scenarios compared with baseline climate, all climate change scenarios projected significantly earlier seeding dates than those currently used. Compared to the baseline conditions, there is no reduction in grain yield because precipitation increases during sensitive growth stages of wheat, suggesting that there is potential to shift seeding to an earlier date. The average advancement of seeding dates varied among sites and chosen scenarios. The Swift Current (south-west) site has the highest potential for earlier seeding (7 to 11 days) whereas such advancement was small in the Melfort (north-east, 2 to 4 days) region.

**Conclusions/significance:**

The extent of projected climate change in Saskatchewan indicates that growers in this region have the potential of earlier seeding. The results obtained in this study may be used for adaptation assessments of seeding dates under possible climate change to mitigate the impact of potential warming.

## Introduction

The date of seeding is an important decision for wheat (*Triticum aestivum* L.) cultivation in Saskatchewan, Canada, since it has a significant impact on the timing of certain stages of phenological development, such as heading and ripening. This can have a profound impact on the damage the plants experience from adverse weather conditions during the growing season, or late season events such as a killing frost (in fall). Khan et al. [1] pointed out that the seeding date of spring wheat is more important than seeding rate for achieving greatest yields. For achieve high yields, therefore, growers must seed as early as possible during suitable weather conditions in the spring when the whole crop is not at sensitive stages when frost may occur. In addition to weather conditions, the soil conditions can also be a key factor for seeding operations. In particular, soil water content and snow cover are assumed to be the main factors affecting seeding date [2], [3], as wet soil may limit the access of seeding equipment to the field.

Using a four year seeding date trial, Gan et al. [4] found that seeding 10 to 12 days earlier than normal could increase grain yield of spring wheat near Swift Current Saskatchewan, Canada. This may be due to lower water stress during anthesis period. By using the concept of “seeding eras” classified by Major et al. [5] and the recent adoption of no-till and continuous cropping, there is a huge potential for early seeding under current and future conditions. With equipment and acreages getting larger, the need for earlier seeding becomes a necessity to ensure that all the land is seeded in a timely manner. These studies offer an opportunity to seek the potential of earlier seeding dates especially considering climate change. However, it is still unknown how early the farmers can seed with minimal risk of spring frost damage and have the land still trafficable while maximizing yields. To confirm the possibility of earlier seeding, long term analysis of a consistent source of seeding dates is required. However, such a requirement is hard to satisfy due to lack of consistency in data collection and tabulation methods.

To obtain a feasible seeding period, many empirical models were developed based on observed seeding dates. Bootsma and Suzuki [6] built a single linear model using average daily mean and mean maximum air temperature as variables to estimate the optimum seeding period. Bootsma et al. [7] further refined the model of Bootsma and Suzuki [6] to obtain a better regression relationship between predicted and observed seeding dates. Major et al. [5] provided a best-fit model of nonlinear relationships through the use of neural networks to predict seeding dates more close to the observed ones. These are very complex with numerous inputs and little user control, making it too difficult to be widely used. Another method of modelling seeding date, proposed by Bootsma and De Jong [3] was to estimate seeding dates of spring wheat with selected environmental criteria and then compare it with observed data. This method considers five criteria and requires that they all be met for 10 days (not necessarily consecutive) prior to seeding: 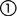
Daily precipitation <2.5 mm; 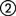 The snow cover <10 mm for the day; 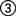 (¾Tmax+¼Tmin) >7°C, where Tmax and Tmin are daily maximum and minimum air temperature; 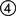 The soil moisture at the top 5% of the soil profile must not exceed 90% Available Water Holding Capacity (AWHC) and 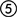 the next 7.5% of the profile must not exceed 95% AWHC. McGinn et al. [8] found that the method developed by Bootsma and DeJong [3] underpredicted the seeding dates at Swift Current, Saskatchewan. In order to get closer to the observed data, they adjusted the criteria such that the daily average temperature (½Tmax+½ Tmin) must exceed 10°C and soil water in the top two soil zones must be less than 90% AWC. The adjusted air temperature and soil moisture criteria, combined with the same daily precipitation and snow cover criteria as Bootsma and DeJong [3] were to be met for 10 days for seeding to occur.

The cited studies all had similar conclusions, namely, that to create a relationship between these criteria and seeding dates, air temperature must exceed a certain value and precipitation and soil moisture must be below certain values for a period of days before seeding occurs. The predicted seeding dates of these models provided considerable information on seeding practices. However, those models aimed to match the observed seeding dates, not to achieve higher yields. The recorded seeding dates only indicate days when seeding occurred as other factors could affect the actual seeding date in a farm. Emphasising a significant relationship between actual and predicted seeding dates eliminates other earlier days that may have been suitable for seeding. In addition, the seeding dates estimated by these empirical models were not connected with yield response.

Inability to control and manipulate other contributing factors in the field makes using traditional field experiments and these empirical models difficult to investigate the effects of earlier seeding. Our previous field phenology study with the widely well-used crop model DSSAT-CSM (Decision Support System for Agrotechnology Transfer - Cropping System Model) [9], [10] provided a basis in this study to model the early seeding dates in different agricultural districts across Saskatchewan. Since previous studies have shown that the DSSAT-CSM model could accurately predict measurements of crop growth in the region, we expect that this model can be used to investigate the effects of early seeding on crop growth and yield. Important attributes of this approach are the ability to freely manipulate seeding periods as desired and to observe the response, which can be difficult to measure experimentally. The objective of this study is to estimate the eariest seeding dates under baseline climate and projected future climate in Saskatchewan, Canada.

## Materials and Methods

### Study sites and crop

**Figure 1:**
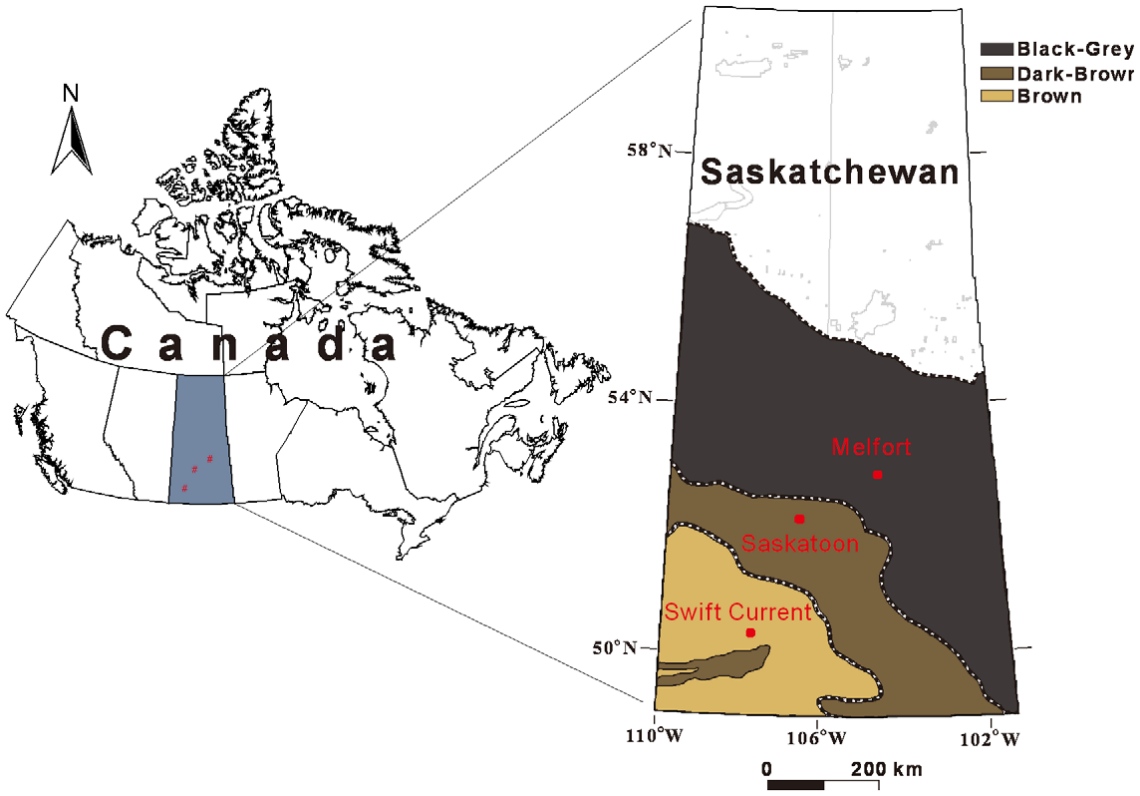
Map of Saskatchewan in Canada, showing major soil zones, and locations of the sites used in this study.

Swift Current, Saskatoon and Melfort, were selected for this study (Fig. 1) and are representative of the brown, dark brown and grey-black soil zones of Saskatchewan, respectively. These sites span an increasing moisture gradient from south-west to northeast, with growing season (May to August) precipitation totals increasing from 188 mm at Swift Current, 202 mm at Saskatoon and 233 mm at Melfort [11]. The selection of these sites takes both the soil and climate into consideration.

Because spring wheat is grown in all crop districts and also because it accounts for almost 70% of the total wheat production in this study area [12], it was selected as the reference crop.

### The DSSAT-CSM Model

The DSSAT-CSM, a widely used process-based modelling package [13], was selected for simulating a wheat production system. This model simulates wheat grain and biomass yield reasonably well in western Canada [10], [14], [15]. The model requires input parameters describing crop and soil characteristics as well as daily weather data. The latter include maximum and minimum temperature, global solar radiation and precipitation, which in the present case were obtained from Meteorological Service of Canada (MSC) [11].

Genetic coefficients of Canadian Prairie Spring wheat class were calibrated with the data collected by Jame and Cutforth [16] and tested using data from the long term New Rotation experiment near Swift Current [17]. In order to predict the long-term effect we used the Sequence Analysis option in the DSSAT-CSM to run the model. Soil property inputs for DSSAT-CSM (organic carbon, clay and silt in percent, pH, soil lower limit of plant extractable soil water, drained upper limit, saturated hydraulic conductivity, saturated upper limit, and bulk density) were observed on site (Table 1). The management used for the simulations was a continuous wheat rotation under no-till with the seeding depth of 5 cm. Nitrogen fertilizer was applied at a rate of 0.1 t ha^−1^at planting time.

**Table 1:** Soil physical properties in the experimental field.

	Depth (cm)	Particle size distribution (%)			Bulk density g cm^−3^	SLLL^a^	SDUL^b^	SSKS^c^ cm d^−1^	SSAT^d^	Organic C (%)	pH
		Clay	Silt	Sand							
	0–15	18	50	31	1.15	0.09	0.28	1.42	0.30	1.8	6.5
	15–30	24	49	26	1.25	0.09	0.28	1.43	0.30	1.5	6.6
	30–45	23	52	24	1.35	0.09	0.28	2.76	0.32	1.3	7.0
Swift Current	45–60	25	33	41	1.4.0	0.10	0.27	4.32	0.34	0.9	7.7
	60–90	32	28	40	1.45	0.10	0.27	5.61	0.34	0.5	7.8
	90–120	33	26	41	1.55	0.10	0.25	3.04	0.34	0.3	8.1
	120–150	33	28	40	1.65	0.10	0.25	3.04	0.34	0.1	8.5
	0–15	60	29	11	1.30	0.11	0.37	7.40	0.59	2.9	7.0
	15–45	60	28	12	1.40	0.11	0.37	7.68	0.57	1.0	7.3
Saskatoon	45–80	60	28	12	1.50	0.11	0.36	5.08	0.53	0.3	7.5
	80–100	60	37	3	1.50	0.11	0.36	1.38	0.53	0.3	8.0
	0–19	50	40	10	1.20	0.20	0.45	0.34	0.55	4.5	6.8
Melfort	19–58	49	44	7	1.30	0.20	0.46	0.29	0.51	1.3	7.0
	58–100	52	45	3	1.30	0.20	0.46	0.25	0.51	0.5	8.0

a Lower limit of plant extractable soil water (cm^3^ cm^−3^).

b Drained upper limit (cm^3^ cm^−3^).

c Saturated hydraulic conductivity cm d^−1^.

d Saturated upper limit (cm^3^ cm^−3^).

### Seeding dates modelling

Four selected environmental criteria (shown in Table 2) were used to restrict the seeding date. The criterion of daily precipitation, snow on ground and soil moisture (top 5 cm) was kept the same as Bootsma and De Jong [3] and McGinn et al. [8]. However, a more practical temperature criterion for the Canadian Prairies (Tmax>10°C) (Stewart Brandt, personal communication) was selected instead of criteria that was used to fit observed seeding dates. Only the top soil (5 cm) moisture was used as a restriction of equipment access. This is not only because soil moisture in many parts of the Prairies in early spring is well below field capacity [18] but also because of better seeding equipment that can allow seeding even if the soil moisture at the deeper depth is equal to 95% soil water capacity (Stewart Brandt, personal communication). Seeding was predicted when conditions met the entire selected criterion simultaneously for four consecutive days.

**Table 2:** Criteria used in estimating seeding dates of spring wheat.

Variable	Criterion
Daily maximum air temperature	>10°C
Daily precipitation	<2 mm
Snow on ground	<10 mm
Soil moisture	<0.9 soil water capacity

The daily maximum air temperature and precipitation were obtained from MSC. The soil moisture was simulated by DSSAT model. Since historic soil water content at each site was unknown, the model was initialized to start five years prior to the period of analysis. A spin-up period of five years was found to be adequate to provide the model with sufficient time for the soil water values to stabilize and not be affected by the initial input values. Through repeated wetting and drying of soils over time, the water content becomes less dependent on the initial estimates [9].

To get the initial conditions (such as soil moisture and snow cover) that are not available from observations, an initial (first) run of the DSSAT model was conducted. It is possible to run only a water balance sub-model with no crop, which is often the case when there is a wheat-fallow rotation system. However, the modelling result with a crop will produce more vivid initial conditions that are closer to the true condition. For this initial run, 15, 20 and 25 March were chosen as a fixed seeding dates in Swift Current, Saskatoon and Melfort, respectively. While those fixed days are a rough approximation of seeding dates, those dates are not expected to have a significant effect on soil moisture during the first few weeks of seedling emergence and early crop growth. During this time, a crop in its very early stages will not influence the water use amounts to any great extent since the main source of water removal would be through evaporation from the soil surface rather than transpiration from the crop. Following the initial model run, the daily spring soil moisture contents were applied to the seeding date criteria.

Before prediction of seeding date, the initial modelling day should be set in advance of expected seeding since the criteria may be met at a time (such as the beginning of March) where the spring wheat can be killed by frost in the following days. To avoid such a circumstance, Bootsma and DeJong [3] selected 15 April as initial day of modelling. This may have been suitable for their study sites. However, the initial day may vary from place to place and time to time due to spatial and temporal variability of weather conditions. A reasonable determination of the initial modelling data should be set according to long term weather records, so the crop has a lower probability of suffering a killing frost in spring. In our study, we set −8°C as a standard of last killing frost in the spring based on previous studies on spring wheat [19]. The lower and upper deciles (10% and 90% probability) of last killing frost were calculated according to the baseline and projected climate. According to the qualitative description provided by the Intergovernmental Panel on Climate Change (IPCC) [20], an event with a probability <10% is very unlikely to occur. In addition, 10% risk is also used as a criterion for crop insurance companies in western Canada [21]. Therefore, this characteristic of probability distribution can effectively describe the probability of killing frost, which is a key characteristic to evaluate for avoidance of agroclimatic risk. However, the temperature of killing frost may be higher or lower depending on the hardening process. In the case of hardening, the prior sequence of temperature events is important when determining the killing frost temperature, a complicated process beyond the scope of this paper.

### Baseline and Climate Change Scenarios

Thirty years of current climate data are normally used in developing a baseline climate scenario [22]. A 30 year period is considered adequate to include a good representation of wet, dry, warm, or cool periods. Selecting a recent 30 year period is preferred because it not only represents the current climate, but also, in most cases, has the most accurate data. In this study, the climate database for these three sites were derived from historic databases between 1961 and 1990 and consisted of daily maximum and minimum air temperature, precipitation and solar radiation. Daily maximum and minimum air temperatures and precipitation were obtained from weather stations located on or nearby the research sites [11]. Daily solar radiation was calculated using the Mountain Climate Simulator [23].

Four climate databases that included a baseline (treated as historic weather climate during the period of 1961–1990) and three climate change scenarios in 2050s (2040–2069) were developed by a Canadian global climate model (CGCM3) ([24], [25] with the forcing of three greenhouse gas (GHG) emission scenarios (i.e., IPCC SRES A2, A1B and B1) [26]. Synthetic 300-yr weather data were generated by AAFC Stochastic Weather Generator (AAFC-WG) for the baseline period and for each scenario [27]. The three commonly used emission scenarios A2, A1B and B1 were used in order of greatest greenhouse gas emissions to least emissions by the end of the century [20]. Qian et al. [28] compared extremes between observed weather and synthetic data. They found the AAFC-WG was capable of reproducing extremes. Therefore, we used the synthetic baseline data instead of real historical weather recording to represent current climate because it only mimics observed weather data on concerned statistical properties and the synthetic data may be more matchable when compared with climate change scenarios [28]. These generated data were used to predict the climate effect on wheat production using the DSSAT-CSM model. Qian et al. [28] found that crop model simulations with 30-yr observed and 300-yr synthetic weather data generated by AAFC-WG with parameters calibrated from the same 30-yr observed data, in general and without considering the effect of seeding dates, do not show significant differences, with regard to timing of biomass accumulation, crop maturity date, as well as final biomass and grain yield at maturity.

### Data Analysis and DSSAT model test

Statistical analyses were performed using SAS [29]. Means and standard deviation of synthetic air temperature and precipitation were calculated and compared among baseline and climate change scenarios by PROC MEANS. Mean difference of predicted and calculated variables were compared between scenarios with PROC MIXED.

Two statistical procedures were used to assess the level of agreement between the predicted value and observed data:

- Root mean square error (RMSE):
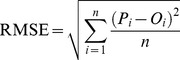
(1)where *P_i_* is the predicted value corresponding to the observed value *O_i_*.
- Index of agreement (*d*) [30]:
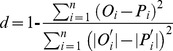
(2)where 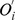 = *O_i_*−*O* and 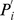 = *P_i_*−*P*; *O* is observed mean and *P* is predicted mean.

The closer the root mean square error (RMSE) is to 0, the more accurate the model is. Index of agreement (*d*) is a measure of the degree of deviation between observed and predicated. The value of *d* is unity when there is a perfect agreement [31].

## Results and Discussion

### Model test

Before application of the DSSAT model, the performance of the DSSAT model was evaluated. A ten year data set (1981–1990) in Swift Current with seeding dates, maturity dates and grain yield were used to test the DSSAT model. The variables tested included maturity date and the final grain yield, as recommended by Hunt et al. [32]. In general, the model gave good predictions of maturity dates and the final grain yield except for the maturity dates in years of 1983, 1986 and 1990 and grain yield in years of 1982 and 1987 (Table 3). Overall, the simulation of maturity dates and grain yield were acceptable with an overall root mean squared error (RMSE) value of 7.3 d and 0.86 t ha^−1^, respectively. Bannayan et al. [33] simulated wheat growth with DSSAT and the model achieved root mean squared errors (RMSEs) of 10.0 d, for maturity dates. Jamieson et al. [34] compared 5 different wheat models in Australia, including CERES-Wheat, and found an RMSE of 0.9 t ha^−1^. Considering that the model doesn't account for pest and disease incidence, the goodness of fit statistics of our results compared favourably to the above reported studies.

**Table 3:** Index of agreement (*d*) of maturity dates and grain yield.

	d-index	
Year	Maturity	Yield
1981	−1.37	0.674
1982	0.945	0.260
1983	0.276	0.662
1984	0.593	0.972
1985	0.960	0.796
1986	0.442	0.521
1987	0.748	0.001
1988	0.992	1.000
1989	0.950	0.959
1990	0.111	0.674

Note: both maturity and yield were measured only once each year.

### Baseline and Climate Change Scenarios

#### Air temperature

**Figure 2:**
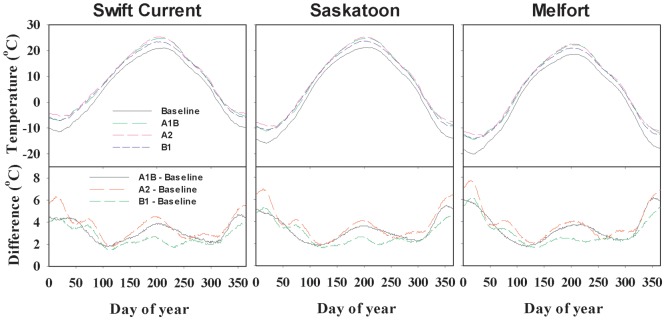
Daily mean temperature under baseline and three scenarios for 2050s, relative to the baseline period 1961–1990, and difference between the baseline and scenarios.

The distribution of air temperature varied among sites under baseline climate. That is, the maximum temperature rose from about 20°C at the beginning of June to about 26°C by the end of July. Compared to the baseline climate, air temperature in all climate scenarios would increase (Fig. 2). The greatest differences in temperature occurred in the winter, followed by summer, and relatively small differences occurred in the spring and fall. Scenario A2 had the greatest increase in annual mean temperature (3.9, 3.6 and 3.5°C for Swift Current, Saskatoon and Melfort, respectively) (Table 4). Annual mean temperature increased from south-west (Swift Current) to north-east (Melfort). The change in pattern and difference between scenarios in daily maximum and minimum air temperatures were similar to that in daily mean temperature (data not shown).

**Table 4:** Mean annual temperature and precipitation total of baseline and three scenarios for 2050s.

Items	Sites	Baseline	A2	A1B	B1
Annual mean temperature (°C)	Swift Current	6.9	10.4 (3.5)	10.0 (3.1)	9.5 (2.7)
	Saskatoon	5.6	9.2 (3.6)	8.7 (3.1)	8.4 (2.8)
	Melfort	1.1	5.0 (3.9)	4.5 (3.4)	4.1 (3.0)
Annual precipitation total (mm)	Swift Current	332	371 (38.8)	387 (54.7)	369 (37.0)
	Saskatoon	349	394 (44.3)	402 (53.2)	391 (42.0)
	Melfort	431	482 (51.0)	483 (52.6)	469 (38.0)

Values in parentheses indicate the change from baseline values.

#### Precipitation

**Figure 3:**
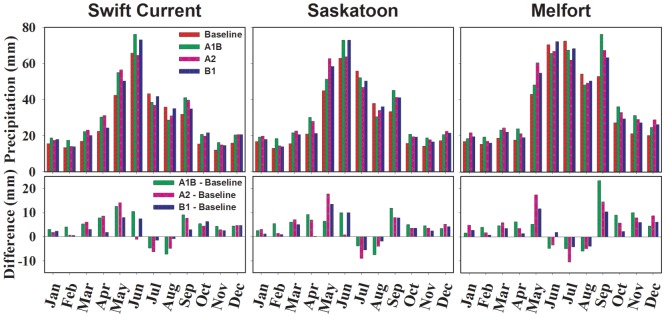
Monthly precipitation under baseline and three scenarios for 2050s, relative to period 1961–1990, and difference between baseline and scenarios.

Under baseline climate, the pattern of precipitation might be fortuitous for spring wheat, with more than 40% falling during the growing season (May to August). The climate change scenarios showed an increase in annual precipitation, compared to the baseline period (Fig. 3 and Table 4) for all sites. The increase in annual precipitation was greatest for A1B and followed by A2 and B1. Total precipitation is predicted to increase 52% above the baseline climate while the precipitation in July and August would decrease for all scenarios. Because precipitation was projected to be less in July and Aug, negative impacts on spring wheat. When precipitation declines, temperatures will increase and negatively impact spring wheat [35]. Precipitation from the A1B scenario was higher than the baseline in every month except from June to August when less rain was projected than the baseline for this period. Scenario A2 was similar to A1B in terms of precipitation distribution except that it was markedly less than A1B in June. Precipitation for scenario B1 was slightly less than that of A1B, except in July and August when B1 had more rain than A1B. Scenario A2 had much more precipitation in Melfort than Swift Current or Saskatoon.

### Seeding dates

**Figure 4:**
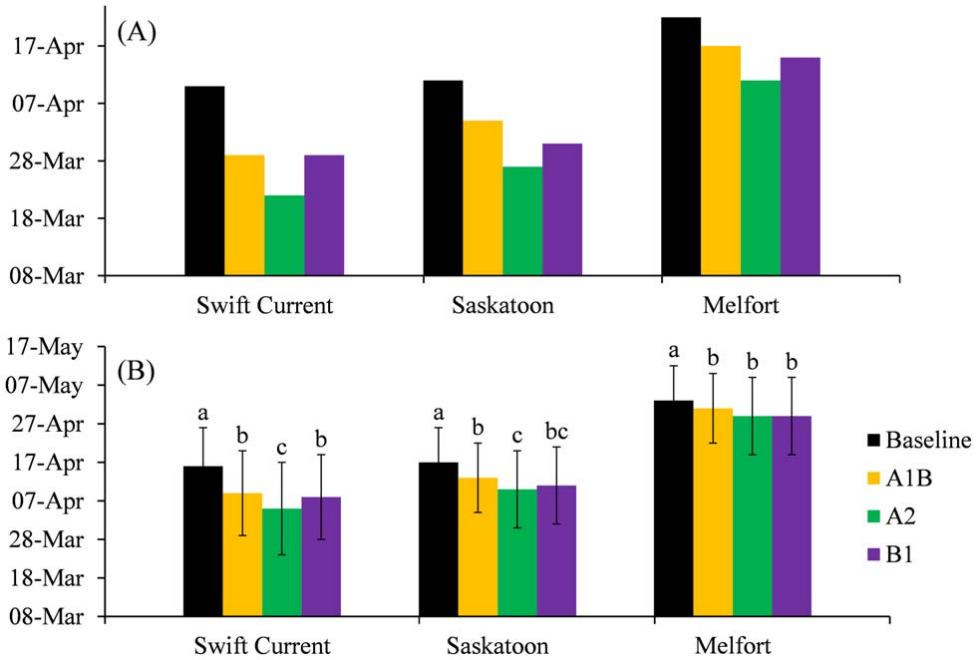
Initial modelling day of baseline and three scenarios for 2050s (A) and averaged seeding dates of baseline and three scenarios for 2050s (B). Within each site, values followed by the same letter are not significantly different at the 0.05 level of probability. Vertical bar indicate standard deviation (days).

The initial dates used in the seeding date modelling (initial modelling day) for Swift Current, Saskatoon and Melfort are shown in Fig. 4A. The initial modelling day was estimated with a less than 10% probability of experiencing a killing frost. Using 100 years of historic weather recording may provide more reliable statistical results. However, due to the “smoothing” effect of long term data, 100 years of data may not reflect the variety of current weather conditions. Therefore, selection of recent 30 year historic weather data may be more reasonable [36].

As shown in Fig. 4A, under a baseline climate, the initial day for modelled seeding dates at Swift Current and Saskatoon were close to each other due to similar annual mean temperature. The initial modelling day for Melfort under the baseline scenario was 11 days later because of its much lower temperature than either Swift Current or Saskatoon. Among the three scenarios, Scenario A1B was closer to the baseline than B1 and A2, which coincides with the temperature distribution pattern.

Simulated baseline seeding dates for spring wheat were averaged for 30 years (Fig. 4B). The earliest averaged seeding date occurred on 16 April (day 106) in Swift Current. Seeding dates were delayed from Swift Current to Melfort, coinciding with lower temperatures influenced by latitude (south-north). The last average seeding occurred in Melfort after 3 May (day 123). The seeding dates under baseline conditions were closer for Swift Current and Saskatoon since their annual mean temperatures were similar.

Due to the temperature increase in the climate change scenarios, the seeding dates were advanced for all sites. For Scenario A2, the earliest seeding date occurred on 5 April (day 95) in Swift Current. For Scenario A1B, the seeding dates were also earliest in Swift Current, and occurred on 9 April (day 99). Again, because of having similar increases in annual mean temperature, there was little difference in seeding dates between Swift Current and Saskatoon in scenarios A1B and B1(Fig. 2 and Fig. 4B).

The results showed that the seeding dates for all sites advance significantly in comparison with the future scenarios and the baseline (Fig. 4B). The change in seeding date between the scenarios and baseline exhibited a south-north trend with the southern areas experiencing a larger increase. In Swift Current, the seeding date was advanced by at least 7 days. Such advancement, however, is possible because i) the seeding date was predicted after the initial modelling day, which has <10% probability of being killed by spring frost; ii) the earlier seeding as recommended by Smith et al. [37] not only ensures a longer growing season, it also reduces the incidence of drought and heat stress during sensitive crop development stages and iii) an acceptable early seeding date should be set based on its corresponding yield, which will be discussed below.

In Melfort, the seeding dates under climate change were close to the baseline seeding dates. Although the increase in annual mean temperature at Melfort was higher, it still wasn't large enough to meet the temperature criterion (10°C), which may explain the minor advance of seeding date at Melfort. Our results are similar to McGinn et al. [8], who also modeled seeding date under climate change scenarios on the Canadian Prairies.

### Phasic Development and precipitation received during critical period

Understanding phasic development of spring wheat is important in matching management decisions and crop inputs with plant development. Although the growth stages under current climate can be recorded, the phenological development and growth in response to environmental factors (soils, weather and management) information is not available in future or projected climate. This problem highlights the importance of using a crop model.

**Figure 5:**
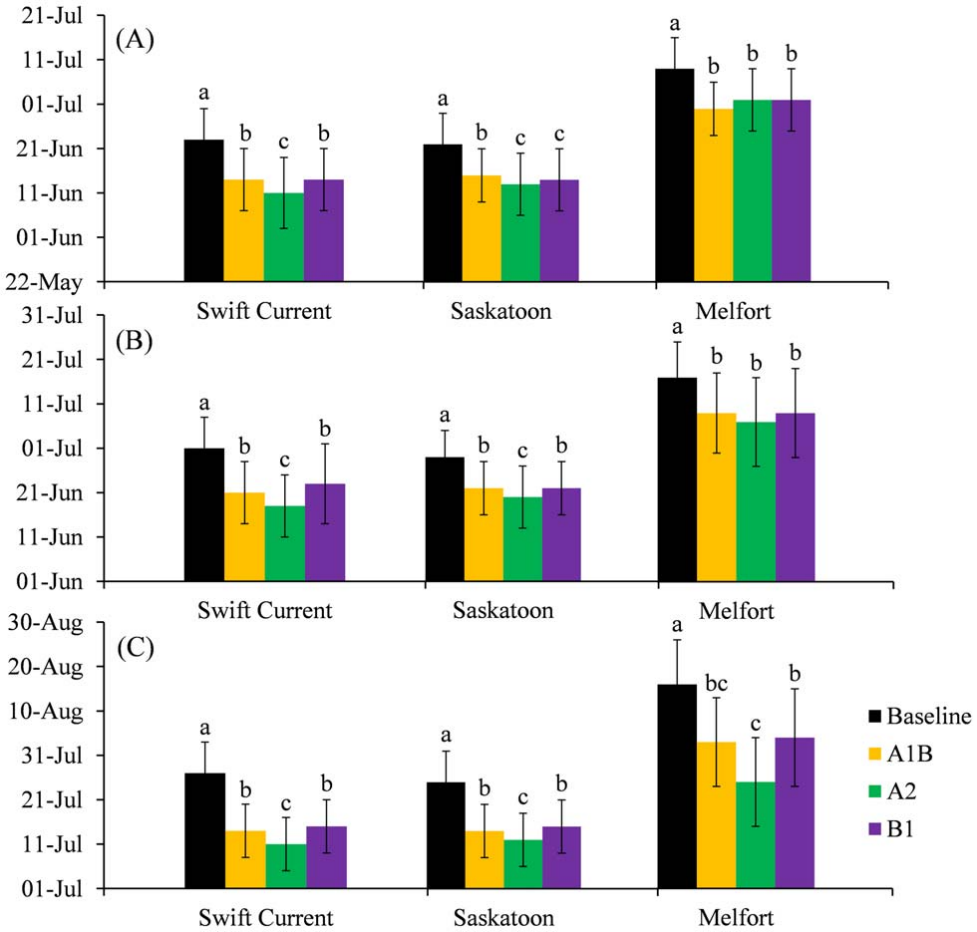
Effects of climate change on phasic development of wheat in (A) anthesis, (B) early dough and (C) maturity. Within each site, values followed by the same letter are not significantly different at the 0.05 level of probability. Vertical bar indicate standard deviation (days).

The predicted seeding dates under all climate change scenarios were earlier than the prediction under the baseline climate for all sites. Because of the earlier seeding and higher temperatures, predicted dates of anthesis and early dough were 7 days earlier than simulations based on baseline climate for all sites (Fig. 5A, B). Crop maturity was advanced by more than 10 days (Fig. 5C). It is noticeable that the RMSE for maturity dates of model evaluation could be due to differences between sites or scenarios. This is understandable since the seeding date, climate and other input parameters are highly variable and thus affect the model output similarly. However, caution is still required since using such process-based crop models is not an error free technique.

The influence of water stress at various growth stages on spring wheat was investigated by Sionit et al. [38] and Mogensen et al. [39]. They found that the stage of anthesis was most sensitive to water stress, followed by the early dough stage. As shown in Table 4 and Fig. 3, although the total annual precipitation will be increased under a projected climate, monthly precipitation in June, July and August will decrease. Historically, average seeding dates are May 9, May 14 and May 15, for Swift Current, Saskatoon and Melfort, respectively. These dates are quite late when compared with our predicted seeding dates. If spring wheat is not seeded earlier than it is currently, it will undergo a severe water stress period under our climate change scenarios.

In order to compare the water stress experienced by spring wheat under baseline climate and projected climate change, we calculated the accumulated precipitation received between seeding and the early dough stage. As shown in Table 5, all scenarios will receive more precipitation during this period when compared with baseline climate due to advanced seeding dates. The increase in precipitation varied among scenarios, of which A1B has highest precipitation in Swift Current and Saskatoon. Compared with Melfort, Swift Current and Saskatoon will receive more precipitation both for baseline and projected climate, which means these two sites have higher yield potential in yield for increase.

**Table 5:** Precipitation received during growth stage from seeding to early dough.

Scenarios	Swift Current	Saskatoon	Melfort
Baseline	128 (45.3)d†	134 (48.5)c	142 (51.4)c
A1B	164 (57.5)a	163 (57.6)a	147 (52.3)b
A2	155 (51.8)b	162 (56.3)a	158 (54.8)a
B1	150 (49.7)c	151 (56.4)b	158 (54.7)a

† Within columns, values followed by the same letter are not significantly different at the 0.05 level of probability.

The growth period can be obtained by calculating the difference between seeding and maturity date. The average simulated growth period for baseline, A1B, A2 and B1 in Swift Current is 102, 103, 104 and 106 days, respectively. This is 5 days longer than our observed (97 days) in Swift Current (1981–1990). Based on a three year cultivar×environment test in a rainfed area, Anderson et al. [40] reported that early seeded long-season cultivars tend to outyielded mid-season cultivars with earlier or late seeding treatment. In a short season environment, Kerr et al. [41] also found that cultivars with longer growth period can achieve higher yield if seeded early. Our results agree with these reports.

### Grain Yield response to simulated seeding dates and climate change

Compared with long-term observations (1961–1990) of maximum grain yield, the grain yield under baseline conditions at Swift Current and Saskatoon were very close to observed values (Table 6). This means the advancement of predicted seeding dates were reasonable under current weather conditions. In Melfort, the simulated grain yield under baseline conditions was much higher than the observed value. The higher grain yield simulated in Melfort was likely due to model insensitivity to disease and pest damage. This site may experiences losses due to insects such as grasshoppers, wheat stem maggots, leaf spots and wire-worms [42], [15].

**Table 6:** Effects of climate change on grain production of wheat.

	Grain Yield t ha^−1^		
Scenario	Swift Current	Saskatoon	Melfort
Baseline	2.16 (0.141)c(1)	2.16 (0.223)b	3.42 (0.334)b
A1B	3.02 (0.239)a	2.85 (0.176)a	4.29 (0.267)a
A2	2.73 (0.180)b	2.83 (0.132)a	4.32 (0.150)a
B1	2.58 (0.223)b	2.79 (0.189)a	4.04 (0.346)a
Historic maximum recording (1960–1990)(2)	2.21	2.24	2.53

^
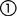
^ Within columns, values followed by the same letter are not significantly different at the 0.05 level of probability.

^
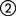
^ From reference [15].

Values in parentheses are the standard deviation.

Chipanshi et al. [15] modeled spring wheat grain yield at the same sites as our study and found the most sensitive parameters influencing grain yield were initial soil moisture, seeding date and thermal condition. In our case initial soil moisture conditions were obtained by 5 years of model training and the thermal condition was determined by weather conditions. Therefore, the grain yield might have been affected by combination of seeding dates and weather condition. The effect of seeding dates in the short-term may not obviously be due to its combination with weather conditions. Through repeated wetting and drying or warm and cold years, the long term effect of seeding date becomes obvious.

The model predicted that all three climate change scenarios had a positive effect on grain yield compared to the baseline (Table 6). The A1B scenario showed the largest yield increases, with 40%, 32% and 25% for Swift Current, Saskatoon and Melfort, respectively due to the projected highest precipitation, while Scenario B1 had the smallest increase for all sites. McGinn et al. [8] and Barklacich and Stewart [43] modeled spring wheat grain yield under climate change scenarios on the Canadian Prairies with similar increased annual mean temperature and total precipitation. They also reported a similar increasing trend in grain yield. Therefore, early seeding for farmers in these areas can benefit from increasing yield but also an employing an important strategy to avoid drought stress in future climate.

## Conclusions

The climatic scenarios in 2040–2069, projected by CGCM3, were used to identify the potential of early seeding dates in agricultural regions on the Canadian Prairies as an adaptation strategy to projected climate change. We predicted earlier seeding dates in a projected future climate for three representative sites located at different latitudes with different soil and weather conditions on the Canadian Prairies. These estimates seemed appropriate as the anticipated yields with earlier seeding dates as predicted by the crop model were similar to or higher than those with actual seeding dates. The average advancement is dependent on the site and the climate scenario. The Swift Current (south-west) site has highest potential for earlier seeding whereas such advancement was small in Melfort (north-east). Our results demonstrated that advancing seeding dates could be helpful in avoiding moisture stress during the sensitive wheat growth stages and to extend the growth period of wheat.

This study was not intended to provide optimal seeding dates but the potential of early ones. The optimum seeding date involves not only reducing unfavorable risk factors (like frost) but also favorable factors like optimum radiation, temperature and moisture. Optimum seeding dates will be investigated in future studies. Caution is required when using process-based crop models to check the simulated seeding dates, since such modelling is not an error free technique and the ability of the model to predict the complicated trait×environment interactions depends on the assumptions made in the model. Nevertheless, such an approach allows us to investigate interactions among seeding date and other environment factors in a way that is not easy in a field experiment. One limitation of the seeding date model in our study is the assumption that the future sowing techniques will be similar to the current era, whereas the seeding date based on “real techniques” in the future may be different. Caution also must be exercised when interpreting the model-simulated results as the effect of heat stress on wheat growth is not well described by the model. Heat stress occurs often in wheat on the Canadian Prairies especially during reproductive growth, which has markedly negative impacts on yield [44]. Under climate change scenarios, the occurrence of heat stress or heat shock may become more frequent. Adaptation measures must be taken with regard to the projected high temperature under climate change. One possible strategy is early seeding. This would allow wheat to mature earlier, avoiding heat shock, which will mostly occur in July (Fig. 2). In addition, the impact of seeding dates in our study depends upon the GHG scenarios used to generate the results. New revisions may produce different impacts of the agroclimate on Prairie agriculture.
